# *T*elmisartan versus Enalap*R*il *I*n heart failure with red*U*ced ejection fraction patients with *M*oderately impaired kidney *F*unctions; randomized controlled trial: “*TRIUMF trial*”

**DOI:** 10.1186/s43044-023-00398-7

**Published:** 2023-08-08

**Authors:** Ahmad Samir, Salma Aboel-Naga, Ahmed Shehata, Magdy Abdelhamid

**Affiliations:** Faculty of Medicine, Cairo University, Cairo, Egypt

**Keywords:** Telmisartan, Enalapril, Heart failure with reduced ejection fraction HFrEF, Chronic kidney disease CKD, Worsening renal function WRF

## Abstract

**Background:**

When heart failure with reduced ejection fraction (HFrEF) and chronic kidney disease (CKD) co-exist, Renin angiotensin-aldosterone system inhibitors (RAASi) are often underutilized for the fear of worsening renal function (WRF). Telmisartan is a RAASi characteristic for a favorable renal profile, although data on its utility in HFrEF is limited. This study aimed to compare efficacy and tolerability of Telmisartan versus Enalapril in patients with HFrEF and CKD.

**Results:**

This study randomized 107 patients with HFrEF and CKD to either Telmisartan (10–80 mg) or Enalapril (5–40 mg) daily. The achieved RAASi dose, dose reductions (DR) or dis-continuation (DC), death/Heart failure rehospitalization (HFH), NYHA class and 6MWT were compared at 3- and 6-months. At 3- and 6-months, 93.5% versus 68.6% and 95.2% versus 72.9% were maintaining ≥ 50% of the target dose in the Telmisartan- versus Enalapril-group, respectively. Despite the higher achieved dose by 3- and 6-months, Telmisartan versus Enalapril was associated with less WRF (6.4% vs. 22.9%, *p* = 0.022 and 7.3% vs. 13.6%, *p* = 0.28) and fewer episodes of DR-DC (31.9% vs. 55.1%, *p* = 0.018 and 35.7% vs. 56.5%, *p* = 0.041), respectively. By the end of the study, there were 5 deaths in each group, yet, HFH occurred in 34.1% versus 55.3%, *p* = 0.035, and NYHA class changed by − 1 [− 2, 0] versus 0 [− 1, 1], *p* = 0.017 in Telmisartan- versus Enalapril patients, respectively. Within-group results showed improvement in 6MWT in Telmisartan-, and increase in diuretic requirements in Enalapril-group.

**Conclusions:**

In patients with HFrEF and CKD, Telmisartan was better tolerated to uptitrate, caused less WRF, less HFH and showed better functional improvement compared to Enalapril.

*Clinical trial registration* This study was prospectively registered on clinicaltrials.gov, with registration number (NCT04736329).

## Background

Heart failure (HF) represents a global public health burden. It affects an estimated population of 64 million people worldwide and is associated with significant morbidity and mortality [1, 2]. The HF syndrome results in decreased cardiac output with subsequent activation of neuro-hormonal compensatory mechanisms, mainly the Renin Angiotensin Aldosterone System (RAAS) and the Sympathetic Nervous System (SNS) [3]. Despite initial usefulness, uncontrolled RAAS activation enhances several deleterious effects including renal sodium reabsorption, fluid retention, worsening congestion, arteriolar vasoconstriction, catecholamines release with overstimulation of the SNS, adverse cardiac remodeling and myocardial fibrosis, all ending into significant worsening of HF prognosis [3, 4]. Hence, RAAS inhibitors (RAASi) are fundamental in the medical management of HF with reduced ejection fraction (HFrEF) [1, 2].

Worsening renal function (WRF) and hyperkalemia are frequent drivers for under prescribing RAASi or failing to reach the target doses. This issue is particularly relevant when HFrEF patients have concomitant chronic kidney disease (CKD) [5]. HF and CKD share several common risk factors and therefore commonly co-exist, with one of them begetting, complicating and exacerbating the other. It is estimated that ≥ 50% of chronic HF patients had estimated Glomerular Filtration Rate (eGFR) of < 60 ml/min/1.73 m^2^, conversely, cardiovascular disease (CVD) remains the leading cause of death in CKD patients [6, 7]. Nevertheless, for the fear of WRF and hyperkalemia, a large proportion of HFrEF patients with CKD are deprived from the cardioprotective and the renoprotective long-term benefits of RAASi [5].

Telmisartan is an angiotensin receptor blocker (ARB), characterized by being primarily excreted via hepatic rather than renal pathway. Provisional experiences with Telmisartan from prior studies suggest better tolerability compared to other RAASi in the management of CVD in CKD patients [8–10].

On these grounds, this study was designed to evaluate the efficacy, safety and tolerability of Telmisartan in HFrEF patients with concomitant moderate renal dysfunction, compared to the standard Angiotensin converting enzyme inhibitor (ACEi) “Enalapril,” in a randomized controlled fashion.

## Methods

This was a prospective, open-label, randomized, controlled, trial that was conducted in Cairo University hospitals during the period from February 2021 to October 2022. The study protocol was approved by the research ethics committee, Faculty of Medicine, Cairo University (MS-352-2020).

The study recruited 107 HFrEF patients with concomitant CKD who met the eligibility criteria after providing a written informed consent. Inclusion criteria comprised: (1) chronic HF with New York Heart Association (NYHA) class II, III or IV, with established diagnosis of HFrEF for ≥ 6 months; (2) CKD with established diagnosis for ≥ 3 months with entry eGFR of 40–60 ml/min/1.73 m^2^; (3) inability to sustain regular Angiotensin Receptor Neprilysin Inhibitor (ARNI) therapy for financial or medical causes; and (4) age between 18-and-80 years. Exclusion criteria included: (1) clinical contraindications to ACEi therapy (i.e., angioneurotic edema on previous exposure or significant bilateral renal artery stenosis); or (2) refusal to participate in the study or withdrawal of the consent at any stage.

In consistence with the clinical guidelines and the universal definition of heart failure, HFrEF was defined as the clinical syndrome of heart failure with stable left ventricular ejection fraction (LVEF) < 40% assessed via transthoracic echocardiography (TTE) by Bi-plane Simpson’s technique [1, 2, 11]. Moderate renal impairment was defined as stable eGFR at the study entry in the range of ≥ 40 to < 60 ml/min/1.73 m^2^. Estimated GFR was calculated by the Modification of Diet in Renal Disease (MDRD-4) equation with consideration of patients’ age, sex, weight and serum creatinine [12].

### Groups allocation

Eligible HFrEF patients were randomized into two equal groups using web-based randomization table and closed envelopes system. Group 1 received Telmisartan (between 10 mg o.d. and − 40 mg b.i.d) as tolerated, while group 2 received Enalapril (between 2.5 mg b.i.d and 20 mg b.i.d) as tolerated. Patients who were already receiving regular RAASi agents (ACEi or ARB) were switched to a starting RAASi dose that is (at least) equivalent to their original agent/dose. Conveniently, total daily doses of 40 mg Enalapril, 80 mg Telmisartan, 10 mg Ramipril or 160 mg Valsartan were considered equivalent to 100% of the recommended target dose, and fractions of these doses were equated accordingly.

Patients who were not on any ACEi or ARBs were started on the smallest dose of the study drug then uptitrated gradually to the recommended or the maximum tolerated dose. Worth mentioning that ability to sustain stable ARNI therapy was re-evaluated during recruitment, and if presumed possible, was considered an exclusion criterion to recruitment. Also, for lack of solid evidence about the maximum Telmisartan dose in HFrEF; in patients with blood pressure (BP) persistently > 140/90 mmHg, it was allowed to increase Telmisartan dose to a maximum of 80 mg b.i.d if not limited by hyperkalemia and/or WRF, while still considered as 100% of the target RAASi dose.

### Upon recruitment

All the study cohort had their basic medical therapy revised. Apart from RAASi, HFrEF therapies with proven survival benefit (namely, Beta-blockers (BB), Mineralocorticoid antagonist (MRA) and Sodium glucose cotransporter type 2 inhibitors (SGLT2i)) were tabulated indicating prevalence of use and the used agent and dose. For standardization, HFrEF therapies were also expressed as percent from the guidelines recommended target dose, (i.e., Bisoprolol 10 mg, Nebivolol 10 mg, Metoprolol 200 mg or Carvedilol 50 mg represented 100% of the recommended BB daily dose). Similarly, oral loop diuretics were tabulated indicating agent and dose. A standardized loop diuretic dose was calculated in Frusemide equivalent dose (i.e., counting Bumetanide 1 mg, or Torsemide 20 mg equivalent to Frusemide 40 mg).

#### Study work-up

Baseline clinical, laboratory and echocardiographic data were collected upon patients’ recruitment, then formally repeated at 3-months and 6-months of follow-up. HF-related symptoms were represented in NYHA classification [1]. NYHA class shifts through the various check-points of the study (baseline, at 3- and 6-months) were represented as +ve or −ve scores for deterioration or improvement, respectively, (i.e., improvement from NYHA III to NYHA I is represented as “− 2”, while deterioration from NYHA II to NYHA III is represented as “+ 1”). 

Effort tolerance was assessed semi-quantitatively by performing a six-minute walk test (6MWT) according to the American Thoracic Society instructions [13], utilizing the 25-m department corridor.

Periodic clinical assessment included evaluation of blood pressure (BP) sitting and 3-min after standing, heart rate, signs suggestive of systemic and/or pulmonary congestion. Orthostatic hypotension was reported when there was a drop between sitting and 3-min standing of > 20 or > 10 mmHg in systolic or diastolic BP measurements, respectively [14]. Orthostatic hypotension was considered as a red flag, mandating consideration of RAASi dose reduction (DR) or transient discontinuation (DC) if other causes were excluded.

Besides clinical evaluation, periodic assessments entailed examination of basic laboratory parameters, particularly serum urea and creatinine, serum electrolytes (including sodium, potassium and magnesium). Detailed TTE assessment of left ventricular (LV) dimensions and function was performed at baseline and 6-months according to American society of echocardiography and the European association of cardiovascular imaging consensus for cardiac chamber quantification [15].

Heart failure rehospitalization (HFH) was defined as clinical decompensation of previously stabilized HF judged to indicate intravenous HF therapies. Rehospitalization for reasons other than decompensated HF, was tabulated but not counted in the study end-points. During hospitalizations, kidney function and serum electrolytes' levels were assessed daily or more frequently if clinically indicated, while for out-patients, these were performed with the scheduled clinical visits every 10–15 days.

### Management of HFrEF therapies through-out the study

As per study protocol, both groups were equally prescribed all other guidelines directed medical therapy (GDMT) for HFrEF, and doses were periodically evaluated if uptitration was considered safely possible. The ability to maintain guidelines-directed doses of foundational HFrEF therapies (specifically BB and MRA) were reported. Also, clinically-judged doses of loop diuretics (expressed in Frusemide dose equivalent) were updated on every clinical visit.

Concerning RAASi, patients were evaluated periodically to uptitrate the study drug if feasible. Conversely, any mandated DR or transient DC through the study period were meticulously reported as well as their primary reason (i.e., intolerable rise in serum creatinine, profound hyperkalemia, symptomatic or orthostatic hypotension, or signs of hemodynamic instability). The composite of DR-DC events was evaluated as a surrogate to drug tolerance.

Dose management of the randomization drug (Telmisartan vs. Enalapril) with respect to the changes in kidney functions followed the expert opinion statement of the French Society of Cardiology [16]. After meticulous patient assessment, including volume status, BP and potential drug-drug interaction to identify and correct any reversible causes, it was acceptable to presume the same RAASi dose if serum creatinine increased by less than 50% of the baseline value, while the dose was reduced by half (i.e., DR) when the rise was 50-to 100% of the baseline value. However, transient DC was triggered when serum creatinine increasesd by ≥ 100% from baseline, till it wanes off [16]. Another indication for DR or DC was significant hyperkalemia (defined as > 5.5 mEq/L) on 2 subsequent days, as per guidelines’ recommendations [1, 2].

### Worsening renal function

Because of lack of standardized definition, WRF had been identified in previous studies when there is absolute increase in serum creatinine from prior assay by ≥ 0.3 mg/d [17, 18], or ≥ 0.5 mg/dL [19, 20], or when there is a relative drop by ≥ 20% in eGFR from baseline [21–23]. Data strongly support that in patients with pre-existing CKD, the definition respecting baseline eGFR and considering a relative decline by ≥ 20% as a diagnostic threshold is better correlated with clinical outcomes [21, 22, 24]. Nevertheless, the investigators resorted to evaluate WRF by all the three validated definitions and express them in the results.

The study objectives were to compare Telmisartan versus Enalapril in regard to the following key components:

- Efficacy
  1. Reduction in the composite of all-cause death and HFH,
  2. Improvement in NYHA class and 6MWT from baseline;
- Safety and tolerability
  1. Ability to safely maintain > 50% of the recommended RAASi dose,
  2. Rates of study-drug DR-DC, as surrogate for drug tolerance,
  3. Prevalence of tolerating other foundational HFrEF therapies (mainly MRAs and BB).

### Statistical analysis

Data were verified, coded then anonymized by the investigators before it was sent to the statisticians. Statistical analysis was conducted using IBM- Statistical Package for the Social Sciences for windows, version 24.0 (IBM-SPSS Inc., Chicago, IL, USA). Descriptive statistics were represented as means ± standard deviations (SD) or medians and interquartile ranges [IQR] for continuous variables, while as frequencies and percentages for categorical variables. For categorical variables, Chi square or Fisher’s Exact tests were used to compare frequencies and McNemar’s test for repeated measures. While for continuous variables, independent *t*-test analysis was carried out to compare the means of normally distributed data, while Mann–Whitney was alternatively used in cases on non-normally distributed data. For continuous variables with more than two categories or repeated measures; repeated measure ANOVA (RM-ANOVA) test was calculated to test the mean differences of the data that follow normal distribution and had repeated measures (between groups, within groups and overall difference). A *P*-value was considered significant when it is less than 0.05.

## Results

**Fig. 1:**
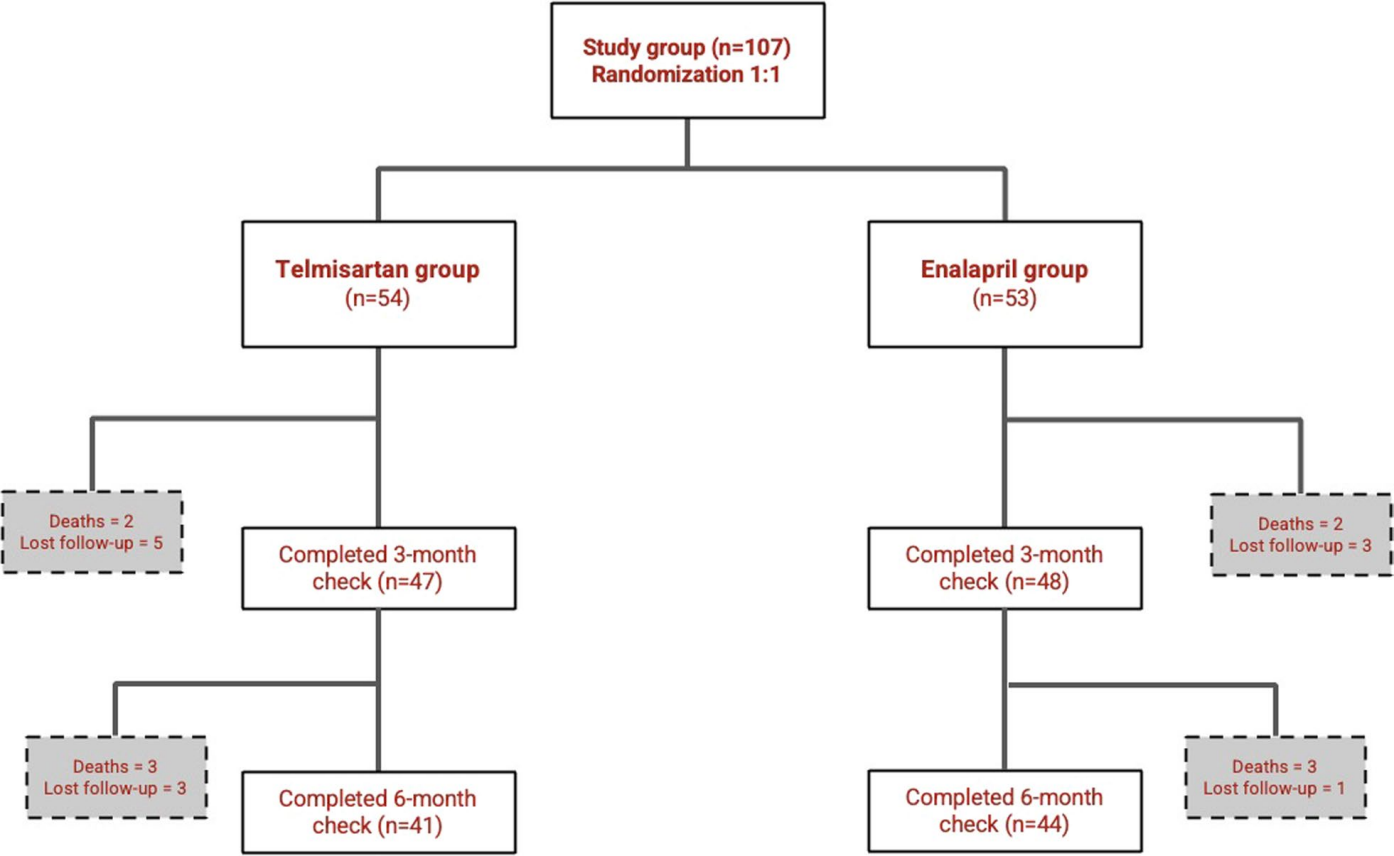
Flow chart for the study recruitment process

This prospective randomized controlled trial recruited 107 eligible HFrEF patients with concomitant moderate CKD as per study protocol. Through the 6-months study period, 12 patients were lost from follow-up and 10 patients died. Deceased patients were calculated in the composite clinical end-point of all-cause death and HFH, but were subtracted from the analyses of clinical- (i.e., NYHA class, 6MWT), laboratory- (i.e., eGFR, WRF) or medications data (study-drug dose, DR-DC) next to their death dates. Flow chart for study recruitment process is demonstrated in Fig. 1.

### Baseline data

Mean age of the study group was 59 ± 9.9 years. Males represented almost 2/3 of the recruited cohort. Baseline features were comparable across the groups except for baseline eGFR which had a small but a significant difference in favor of the Enalapril- over the Telmisartan group.

During recruitment, there were no significant differences between the 2 groups in the standardized doses of diuretics, BB, MRA or SGLT2i. Similarly, the starting doses of Telmisartan and Enalapril were comparable, with median [IQR] of 50% [50–50] of the RAASi target dose in both groups.

Detailed baseline clinical, laboratory and echocardiographic features of the whole study group, and comparison between those randomized to Telmisartan versus Enalapril are demonstrated in Table 1.

**Table 1:** Baseline clinical, laboratory and echocardiographic parameters of the study groups

	Total (*n* = 107)	Telmisartan (*n* = 54)	Enalapril (*n* = 53)	*P*-value*
Age (years)	59.3 ± 9.9	60.13 ± 10.1	58.42 ± 9.7	0.38
Male sex	72 (67.9%)	35 (64.8%)	37 (69.8%)	0.485
Height (cm)	163.98 ± 6.5	163.76 ± 6.1	164.21 ± 6.8	0.725
Weight (kg)	77.21 ± 9.2	76.72 ± 9.6	77.87 ± 8.6	0.736
BMI (Kg/m^2^)	29.04 ± 6.6	29.33 ± 7.4	28.74 ± 5.6	0.654
NYHA				0.204
I–II	64 (59.8%)	35 (64.8%)	29 (54.7%)	
III	25 (23.4%)	11 (20.4%)	14 (26.4%)	
IV	18 (16.8)	8 (14.8%)	10 (18.9%)	
Baseline 6MWT	112.88 ± 74.83	104.91 ± 10.3	121.01 ± 10.1	0.268
Baseline Serum Cr. (mg/dl)	1.68 ± 0.46	1.72 ± 0.4	1.65 ± 0.4	0.441
Baseline eGFR (ml/min/1.73 m^2^)	49.7 ± 7.63	47.91 ± 7.4	51.55 ± 7.5	**0.013**
Baseline serum K (mEq/L)	4.22 ± 0.59	4.27 ± 0.5	4.16 ± 0.6	0.332
Baseline serum albumin	3.4 ± 0.43	3.4 ± 0.5	3.4 ± 0.4	0.529
LV Ejection fraction	32.05 ± 2.2	32.31 ± 2.1	31.77 ± 2.4	0.656
LV EDD	6.34 ± 0.6	6.33 ± 0.6	6.26 ± 0.7	0.626
LV ESD	5.37 ± 0.8	5.27 ± 0.8	5.20 ± 0.9	0.659
TR-Vmax (m/s)	2.70 ± 0.7	2.82 ± 0.7	2.52 ± 0.7	0.162
Starting diuretic dose†	107 ± 117	100 ± 99	108 ± 104	0.503
Starting BB dose ‡	25% ± 22.6	22% ± 20	28% ± 25	0.134
Starting MRA dose ‡	61% ± 26	59% ± 22	63% ± 30	0.548
Starting RAASi dose ‡	50% [50–50]	50% [50–50]	50% [50–50]	0.09

Bold refers to statistically significant *p* value

6MWT—Six-minute walk test; BMI—body mass index; Cr.—creatinine; EDD—end diastolic diameter; eGFR—estimated glomerular filtration rate (by MDRD-4 formula); ESD—end systolic diameter; K—potassium; LV—left ventricular; NYHA—New York heart association; TR-Vmax—Tricuspid regurgitation jet maximum velocity

^*^Representing the comparison of distribution between Telmisartan versus Enalapril groups

^†^Expressed in mean ± standard deviation (SD) of Frusemide equivalents, considering 20 mg of Torsemide, 1 mg of Bumetanide and 40 mg of Frusemide as equivalents

^‡^Expressed as either mean ± SD or median [IQR] percentage of the guidelines recommended target dose

### By the 3-months check-point

Through the first 3 months of the study, maintaining ≥ 50% of the target RAASi dose in the Telmisartan-compared to the Enalapril-group was achieved in 93.5% versus 68.6%, respectively, (*p* = 0.002). The median [IQR] of the utilized dose in the Telmisartan versus Enalapril represented 100% [100–100] versus 50% [25–100] of the target dose, respectively (*p* < 0.001). Additionally, the odds ratio (OR) and 95% confidence interval (CI) for Telmisartan- compared to Enalapril-allocated patients to maintain ≥ 50% of the target dose was 1.31 [1.09–1.57].

Despite the higher achieved RAASi dose, Telmisartan- compared to Enalapril-group had significantly fewer episodes of DR (10.6% vs. 32.7%, *p* = 0.008) and the composite of DR-DC episodes (31.9% vs. 55.1%, *p* = 0.018). There were no differences in serum creatinine, serum potassium or eGFR between the 2 groups. WRF_GFR_ (defined as ≥ 20% drop of the eGFR from the prior assessment) occurred significantly less in the Telmisartan- than Enalapril-group (6.4% vs. 22.9%, *p* = 0.022).

Maintaining BB and MRA therapies at 3-months was nearly similar between the Telmisartan versus the Enalapril group, suggesting acceptable tolerability of other HFrEF foundational therapies. The standardized doses of loop diuretics were numerically higher in the Enalapril- compared to the Telmisartan group, (109 ± 126 vs. 125 ± 113 mg of Frusemide equivalents, *p* = 0.362).

Concerning the clinical endpoints at the 3-months evaluation, rates of all-cause death, of HFH and of the composite of all-cause death/HFH were comparable between the Telmisartan and the Enalapril, reaching 2 (4%) versus 2 (4%), 9 (18.4%) versus 14 (28%) and 11 (22.4%) versus 14 (28%), respectively.

The median change in NYHA class (expressed as + ve or -ve units for deterioration or improvement, respectively) was comparable. The remainder of clinical data from baseline-to-the 3-months assessment are detailed in Table 2.

**Table 2:** Data of baseline-to-3-months period

	Telmisartan	Enalapril	*P*-value
RAASi standardized dose*	100% [100–100]	50% [25–100]	**< 0.001**
Achieving ≥ 50% of the target RAASi dose	93.5%	68.6%	**0.002**
Occurrence of DR	10.6%	32.7%	0.008
Composite of DR-DC	31.9%	55.1%	0.018
Peak serum creatinine	1.68 ± 0.57	1.63 ± 0.50	0.61
Estimated GFR	48.89 ± 12.44	54.04 ± 17.86	0.11
Serum potassium	4.64 ± 0.56	4.57 ± 0.71	0.72
Rate of WRF (1)	14%	20.8%	0.31
Rate of WRF (2)	8.5%	12.5%	0.384
Rate of WRF (3)	6.4%	22.9%	**0.022**
Maintaining MRA therapy	89.4%	87.5%	0.51
Maintaining BB therapy	87.2%	93.8%	0.23
BB standardized dose*	39.4 ± 33.5%	43.2 ± 32.4%	0.569
Loop diuretic standardized dose^†^	109 ± 126	125 ± 113	0.362
Composite of all-cause death and/or HHF	22.4%	28%	0.343
All-cause death	4%	4%	0.684
HFH	18.4%	28%	0.185
NYHA classification at 3-months [*n* (%)]			
I–II	30 (63.8%)	33 (68.8%)	0.785
III	11 (23.4%)	8 (16.7%)	
IV	6 (12.8%)	7 (14.6%)	
NYHA class shifts between baseline-to-3-months	0 [− 1, 0]	0 [− 1, 0]	0.616

Bold refers to statistically significant *p* value

BB—Beta adrenergic blocker; DC—discontinuation; DR—dose reduction; GFR—glomerular filtration rate; HFH—heart failure re-hospitalization; MRA—Mineralocorticoid antagonists; NYHA—New York Heart Association class; RAASi—Renin–angiotensin–aldosterone system inhibitors; WRF—worsening renal function defined as absolute rise in the serum creatinine by 0.3 mg/dl, or by 0.5 mg/dl or as relative decline of the eGFR by ≥ 20% compared to prior assessments in (1), (2) and (3), respectively

^*^Representing percentage from the target dose

^†^Expressed in mean ± standard deviation (SD) of Frusemide equivalents, considering 20 mg of Torsemide, 1 mg of Bumetanide and 40 mg of Frusemide as equivalents

### Between 3 and 6 months

By the 6-months evaluation, 95.2% and 72.9% of the Telmisartan- and the Enalapril-patients were maintaining ≥ 50% of the RAASi target dose, *p* = 0.004, with median [IQR] of the target dose of 100% [100–100] versus 100% [25–100], respectively, (*p* < 0.001). The OR for Telmisartan- compared to Enalapril-allocated patients to maintain ≥ 50% of the target dose by 6-months was 1.31 [95% CI 1.1–1.6].

DR events occurred numerically fewer in the Telmisartan- compared to the Enalapril group in 23.8% versus 39.1%, respectively, *p* = 0.094. However, the composite of DR-DC was significantly less in the Telmisartan- compared to the Enalapril-group, occurring in 35.7% versus 56.5%, respectively, *p* = 0.041. Despite the higher achieved RAASi dose, Telmisartan was associated with almost half the rate of WRF_GFR_ compared to the Enalapril group (7.3% vs. 13.6%), yet without achieving statistical significance.

Rates of new HFH events between 3-to-6 months tended to be fewer in the Telmisartan- compared to Enalapril-group, reaching 7 (15.9%) versus 15 (31.9%), *p* = 0.061. There were 3 new deaths in each group through that period. The composite of all-cause death/HFH showed a trend for fewer events in the Telmisartan compared to Enalapril, with a rate of 8 (18.2%) versus 16 (34%), *p* = 0.069. The total (through the whole 6-months period) rate of all-cause death was comparable (5 cases in each group), while HFH and composite of death/HFH were significantly less in Telmisartan- compared to Enalapril-patients with rates of 15 (34.1%) versus 26 (55.3%), *p* = 0.035 and 18 (36.7%) versus 28 (57.1%), *p* = 0.034, respectively.

By the end of the study period, Telmisartan-group showed significantly better NYHA class shifts (changes from baseline) compared to Enalapril, with a median change of − 1 [− 2, 0] versus 0 [− 1, 1], *p* = 0.017. The distribution of NYHA classes was non-significant between the 2 groups at 6-months, however, the within-group changes (comparing baseline-to-the end of the study period) exhibited a favorable re-distribution only in the Telmisartan that seemed mainly derived from the noticeable reduction in NYHA class IV prevalence, [changing from 14.8 to 7.3% vs. 19 to 25% in the Telmisartan vs. the Enalapril-group, respectively].

Similarly, despite the non-significant between-groups difference in the 6MWT, the within-group changes (by repeated measures ANOVA) pointed to a statistically significant improvement in the Telmisartan group but not in the Enalapril group. Furthermore, the standardized doses of loop diuretics showed nearly stable requirements from baseline to the end of the study in the Telmisartan group contrasted to an obvious progressive rise in the Enalapril group through successive check-points that demonstrated significant within-group differences.

**Fig. 2:**
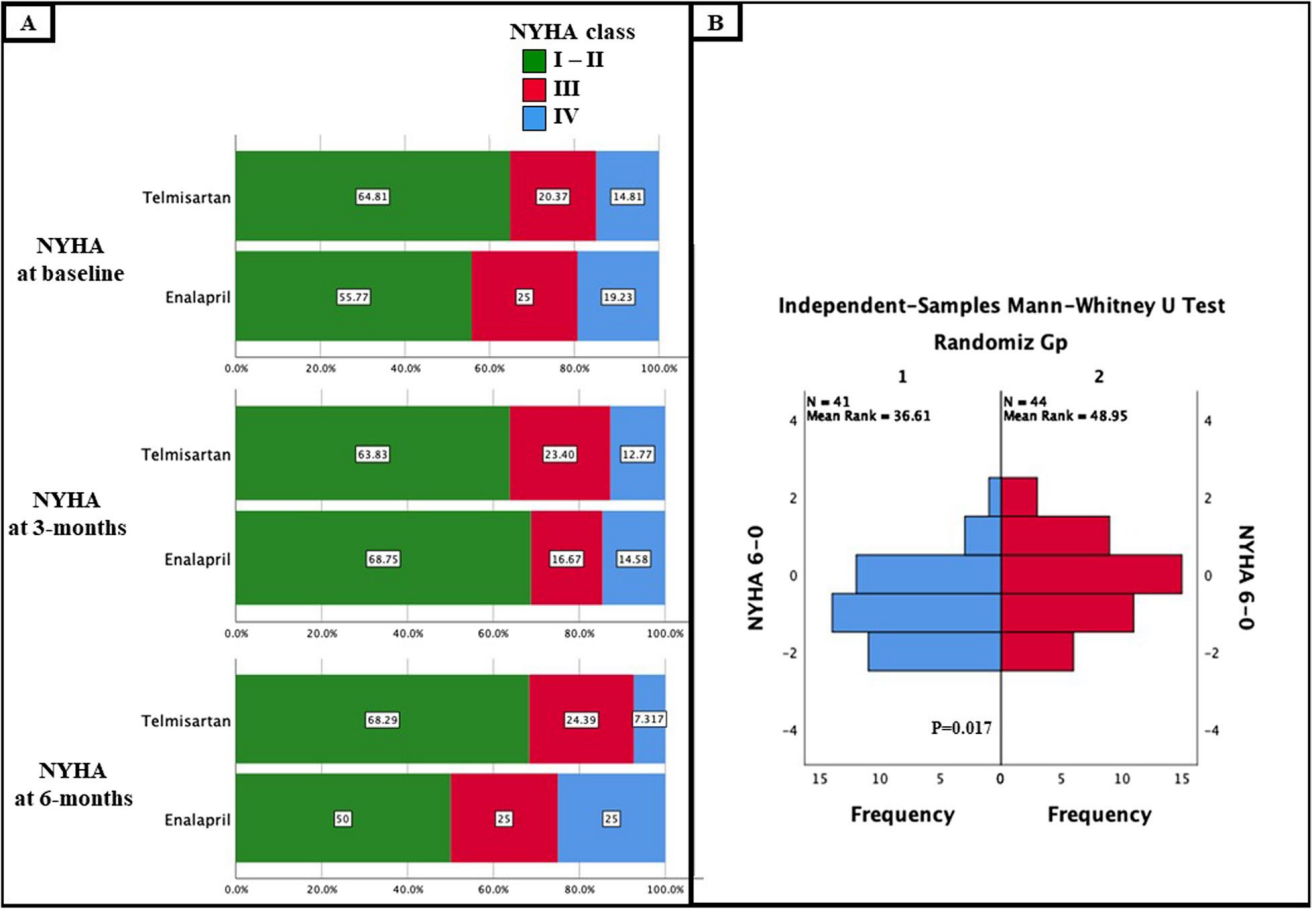
**A** Distribution of NYHA class across the Telmisartan- and Enalapril groups at baseline and by the end of the study. **B** Mann–Whitney testing of the NYHA class shifts through the study period (baseline -to-6 months) expressed as − 1 for each class improvement (i.e., IV-to-III) and + 1 for each class deterioration (i.e., II-to-III)

**Fig. 3:**
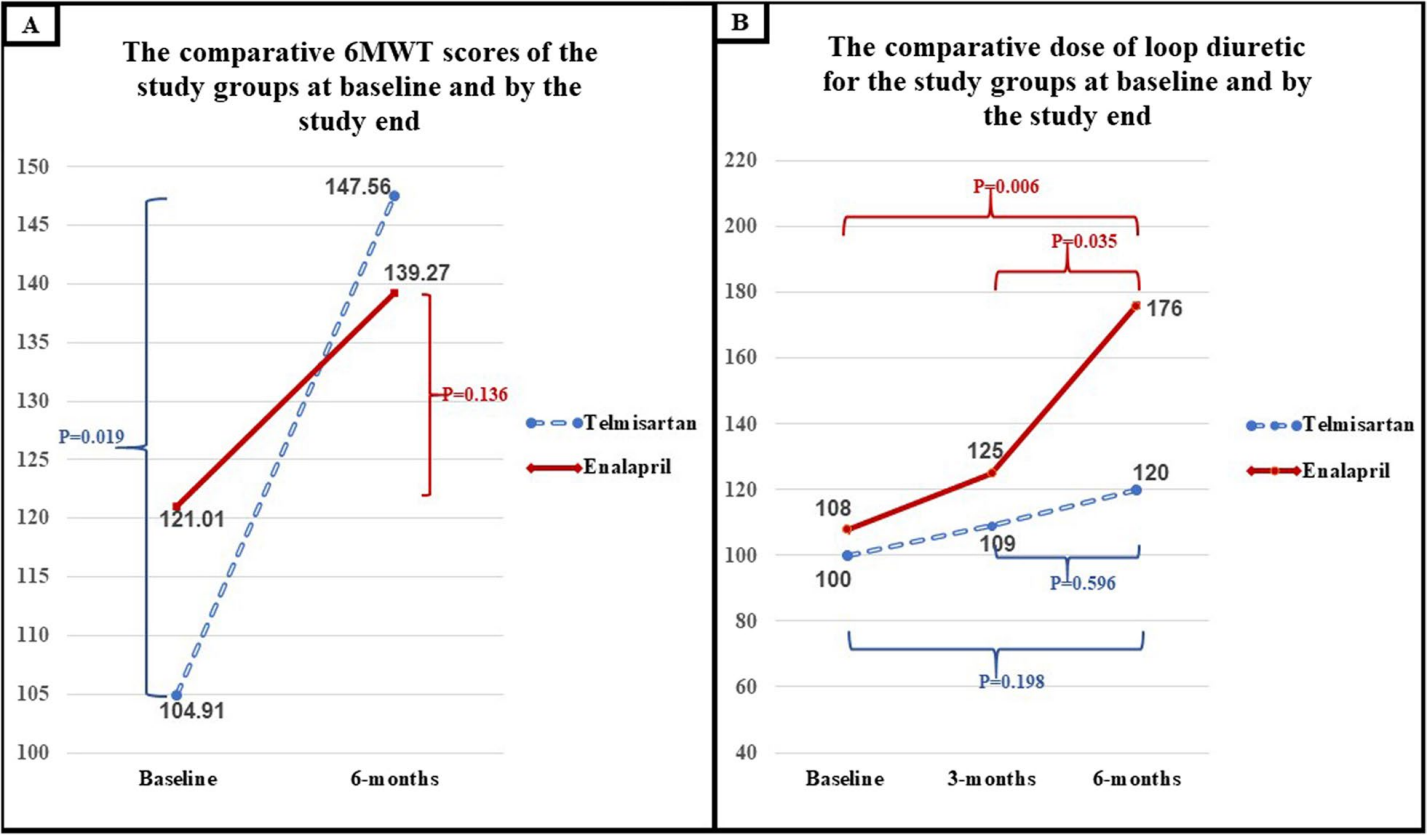
**A** Scores of 6MWT for the 2 groups at baseline and by the end of the study with demonstration of the within-group *p* value. **B** Daily requirements of the standardized loop diuretics dose from baseline to the end of the study demonstrating changes in dose requirements and the p values of within-group differences between baseline-to-6 months and 3-to-6 months

The details of the 6-month data are shown in Tables 3 and 4, and Figs. 2 and 3.

**Table 3:** Data by the 6-months assessment

	Telmisartan	Enalapril	*P*-value
RAASi standardized dose*	100% [100–100]	100% [25–100]	**< 0.001**
Achieving ≥ 50% of the target RAASi dose	95.6%	72.9%	**0.009**
Occurrence of DR	23.8%	39.1%	0.094
Composite of any DR-DC	35.7%	56.5%	**0.041**
Peak serum creatinine	1.70 ± 0.51	1.72 ± 0.69	0.853
Estimated GFR	48.6 ± 10.7	53.9 ± 17.7	0.098
Serum K	4.65 ± 0.59	5.57 ± 0.60	0.558
Rate of WRF (1)	19.5%	25%	0.365
Rate of WRF (2)	9.8%	11.4%	0.546
Rate of WRF (3)	7.3%	13.6%	0.278
Maintaining MRA therapy	93%	84%	0.198
Maintaining BB therapy	93%	86%	0.278
BB standardized dose*	47.9 ± 33.8%	50 ± 37.5%,	0.783
Loop diuretic standardized dose^†^	120 ± 123	176 ± 208	0.143
New composite of all-cause death or HFH	18.2%	34%	0.069
New all cause death	3 (6.8%)	3 (6.4%)	0.63
New HFH	15.9%	25.5%	0.192
NYHA class shifts between baseline-to-6-months	− 1 [− 2, 0]	0 [− 1, 1]	**0.017**

Bold refers to statistically significant *p* value

BB—Beta adrenergic blocker; DC—discontinuation; DR—dose reduction; GFR—glomerular filtration rate; HFH—heart failure re-hospitalization; MRA—Mineralocorticoid antagonists; NYHA—New York Heart Association class; RAASi—Renin–angiotensin–aldosterone system inhibitors; WRF—worsening renal function defined as absolute rise in the serum creatinine by 0.3 mg/dl, or by 0.5 mg/dl or as relative decline of the eGFR by ≥ 20% compared to prior assessments in (1), (2) and (3), respectively

^*^Representing percentage from target dose; † Expressed in mean ± standard deviation (SD) of Frusemide equivalents, considering 20 mg of Torsemide, 1 mg of Bumetanide and 40 mg of Frusemide as equivalents

**Table 4:** Between-groups and within-group differences in NYHA class, 6MWT and required maintenance loop diuretic doses at baseline and by the end of the study

NYHA classification [*n* (%)]		Telmisartan	Enalapril	*Between groups P*-value
Baseline	I–II	35 (64.8%)	29 (55.8%)	0.655
	III	11 (20.4%)	13 (25%)	
	IV	8 (14.8%)	10 (19.2%)	
6-months	I–II	28 (68.3%)	22 (50%)	0.071
	III	10 (24.4%)	11 (25%)	
	IV	3 (7.3%)	11 (25%)	
Within-group P-value*		**0.024**	0.108	

6-MWT [mean ± (SD)]		Telmisartan	Enalapril	*Between groups P*-value
Baseline		104.91 ± 10.3	121.01 ± 10.1	0.268
6-months		147.56 ± 11.6	139.27 ± 15.2	0.668
Within-group P-value*		**0.019**	0.136	

Maintenance loop diuretic dose^†^ [mean ± (SD)]		Telmisartan	Enalapril	*Between groups P*-value
Baseline		100 ± 99	108 ± 104	0.503
6-months		120 ± 123	176 ± 208	0.143
Within-group P-value*		0.198	**0.006**	

Bold refers to statistically significant *p* value

6MWT—6-Minute walk test; NYHA—New York Heart Association class

^*^Highlighting the difference within each group between baseline to the end of the study by McNemar or repeated measures ANOVA testing as appropriate

^†^Expressed in Frusemide equivalents, considering 20 mg of Torsemide, 1 mg of Bumetanide and 40 mg of Frusemide as equivalents

## Discussion

In this randomized controlled study selectively recruiting 107 patients with HFrEF and concomitant CKD who were unable to sustain ARNI therapy, Telmisartan compared to Enalapril was better tolerated, achieved higher percentages of the target dose, was associated with fewer episodes of mandated DR and/or DC, while reducing rates of worsening renal functions. The improved RAAS inhibition in the Telmisartan arm (better tolerability and sustainability), was translated to more favorable changes in the NYHA class and 6MWT and reduction in rehospitalization, compared to the Enalapril-allocated controls.

RAASi are cornerstone in HFrEF management with proven symptoms and survival benefits, hence, are granted class I recommendation in most practice guidelines [1, 2]. In the PARADIGM-HF trial, the ARNI (Sacubitril/Valsartan) showed substantial reduction in the composite of death/rehospitalization when compared to the prototype ACEi, the Enalapril [25]. Since then, ARNI had become the preferred RAASi agent. However, for the significant high monthly cost, sustained ARNI use is underutilized among the indicated cohorts in low-to-mid-income countries, particularly when not fully insured [26]. Moreover, in the PARADIGM-HF trial, after 10,513 initially entered the study, around 20% were filtered out through the roll-in phase mainly because of drug intolerance, ending in 8442 proceeding to the randomization phase [25]. Hence, at least for these 2 main limitations, it is very clear that even in the era of ARNI, the role of conventional and readily affordable RAASi (ACEi/ARBs) in HFrEF management is not over yet.

One of the main identified barriers to RAASi in HFrEF management is the consequent impairment of renal functions, specially in patients with pre-existing renal dysfunction [5]. Acknowledging the intimate overlap of risk factors and injurious agents/conditions for CVD and CKD, one can appreciate the high prevalence of their co-existence, and that one of them can cause and/or exacerbate the other, while the two of them begets worsening of each other [6, 27]. It is well established that CVD is the leading cause of death in CKD patients, while conversely, an eGFR < 60 ml/m/1.73 m^2^ was prevalent in nearly 60% of HF hospitalizations [7].

Importantly, full benefits of RAASi (including ACEi/ARBs/ARNI) on both CV- and renal-outcomes are conditioned by sustaining the doses identified by clinical trials and recommended by guidelines. This belief is supported by multiple trials and registries where HF patients limited to low-dose RAASi demonstrated significantly poorer outcomes including all-cause mortality, all cause hospitalization, CV hospitalization as well as renal outcomes compared to those receiving higher doses [28–30]. In the large CHAMP-HF registry, < 30% of the indicated cohort could achieve the RAASi target dose (including ACEi/ARB/ARNI), while patients sustaining ≥ 50% of the target dose showed significantly better NYHA class and less HFH compared to those receiving lower doses [5].

Hence, from the grounds of registries and daily clinical practice, a large cohort of HFrEF with co-existing CKD are denied the substantial clinical benefit of RAASi. For the fear of WRF, these patients are either completely deprived from-, or at the best, are prescribed low-dose of RAASi [31]. Thereby, a RAASi agent that can be better tolerated in HFrEF with concomitant CKD remains to be a lingering unmet need.

Unlike most ACEi/ARBs that are dependent on renal clearance, Telmisartan is an ARB unique for a predominant hepatic clearance, reducing the risk of drug accumulation or toxicity in patients with CKD [32]. The ONTARGET and TRANSCEND studies demonstrated that Telmisartan is an appealing RAASi in the management of hypertension, CVD and diabetes with end-organ damage, particularly in ACEi intolerant groups [33, 34].

Additionally, Telmisartan was postulated to provide further renal benefits independent form its characteristic predominant hepatic clearance. In a dedicated analysis of the > 25,000 participants in the ONTARGET study, there was significant progressive reduction in proteinuria observed in the Telmisartan arm, with improved renal outcomes by the end of the study [34]. The mechanism of such renoprotective effect is not fully understood, though is suggested to be related to the agonistic effects of Telmisartan on the Peroxisome Proliferator Activated Receptor-Gamma (PPAR-γ) pathway [35].

Although the evidence on Telmisartan in HFrEF remains limited, in the few available studies Telmisartan was found to be at least as good as other “standard” RAASi agents in HF. In a study that had randomized 120 NYHA III patients to receive either Telmisartan or other standard ACEi, besides other GDMT, both arms showed equivalent improvement in LVEF and serum brain natriuretic peptide (BNP) levels after 1-year of therapy [36]. In another double-blinded randomized study recruiting 378 HFrEF patients on stable therapy, both Telmisartan and Enalapril arms showed comparable improvements in effort tolerance, with a numerical reduction in rates of cough complaint in the Telmisartan-arm that did not achieve statistical significance [37]. Notably, these studies were in non-selected HFrEF patients who were able to receive guidelines-recommended doses of RAASi agents, while dedicated studies in the subgroup of HFrEF and concomitant CKD seemed lacking.

In the present study, we have selectively recruited HFrEF patients with concomitant moderate renal impairment who cannot sustain regular ARNI therapy. Moderate renal impairment was defined as entry eGFR of 40–60 ml/min/1.73 m^2^. Baseline features were comparable between the 2 groups except for a small but significant difference in the eGFR in favor of Enalapril-group. Through the 6-months study period, Telmisartan was significantly better tolerated than Enalapril. By the 3- and 6-months check-points, the median utilized dose in the Telmisartan-group achieved 100% [100–100] and 100% [100–100] of the target dose compared to 50% [25–100] and 100% [25–100] in the Enalapril-group, respectively. Better tolerability was well demonstrated by the proportion of patients sustaining ≥ 50% of the target dose, which was 93.5% versus 68.6% by the 3-months, and 95.2% versus 72.9% by the 6-months check-points, for the Telmisartan versus Enalapril, respectively. A cut-off of ≥ 50% of the target dose was considered after it had shown in the CHAMP-HF registry to be associated with significantly better mid- and long-term clinical benefits compared to lower doses [5].

WRF seemed to lack standardization of a diagnostic threshold, with some definitions utilizing an absolute increase in serum creatinine while others considering a relative reduction in the eGFR. Among the most utilized definitions in HF studies was an absolute increase of ≥ 0.3 mg/dL in serum creatinine level within 5 days, compared to the baseline value [17, 18]. Another definition used alternatively the cut-off of absolute increase by ≥ 0.5 mg/dL as the diagnostic threshold [19, 20]. A third definition was later proposed and proved to be a better prognosticator for patients with already pre-existing renal impairment, and instead of an absolute change in serum creatinine, it identified WRF as a drop by ≥ 20% in eGFR from baseline [21–23]. The selective recruitment of patients with renal dysfunction would arguably make the WRF definition relying on a relative drop from the baseline eGFR (WRF_GFR_) more meaningful [23, 38].

In this study, despite the higher achieved RAASi dose in the Telmisartan-group, the rate of WRF_GFR_, was significantly less compared to the Enalapril-group. Moreover, the rates of DR and DC were less in the Telmisartan- compared to Enalapril-group, supporting a better tolerability- and safety-profiles of Telmisartan in this selective HFrEF cohort.

Concerning clinical outcomes through the study period, death events were comparable, however, rates of HFH and of the composite of death/HFH were significantly less in the Telmisartan compared to Enalapril. Indeed, the distribution of NYHA classes between the 2 groups did not achieve statistical significance, however, the within-group distribution at 6-months compared to baseline demonstrated a significant change (improvement) in Telmisartan- but not in Enalapril-group. More interestingly, NYHA class shifts t in the Telmisartan versus Enalapril demonstrated significant improvement by the 6-months- compared to baseline-evaluation, with a median change of − 1 [− 2, 0] versus 0 [− 1, 1], *p* = 0.017. Effort tolerability represented by 6MWT showed similar results to NYHA distribution, with a non-significant between-groups difference, but a significant improvement from baseline to 6-months in the Telmisartan- while not in the Enalapril-group.

Another supportive observation for the favorable clinical outcome was the stable loop diuretic dose in the Telmisartan- in contrast to the progressively increasing requirements in the Enalapril-group. Higher loop diuretic dose was found to be an independent predictor for worse outcomes and increased mortality as demonstrated in a large study recruiting 1354 HFrEF patients, showing adjusted HR of death of 4.0 (95% CI 1.9–8.4) for those requiring > 160 mg compared to those requiring 0-to-40 mg daily equivalent diuretic dose [39].

In fact, there is paucity of RCT-derived evidences on safety and efficacy of RAASi use in patients with advanced kidney disease. According to a recently published state-of-the-art review [40], only one RCT was available evaluating RAASi therapy in HFrEF patients with end-stage kidney disease (ESKD). In that single study, the investigators chose Telmisartan for that selected cohort, and concluded that Telmisartan compared to placebo reduced both; all-cause- and cardiovascular-mortality with HR of 0.51 [0.32–0.82] and 0.42 [0.38–0.61], respectively, with similar rates of DC of the study drug through the ≥ 3-years of the study, supporting an excellent tolerability of Telmisartan in CKD patients [41].

Another important evidence for the utility of Telmisartan in patients with poor tolerability to ACEi comes from a larger (*n* = 5962) study selectively recruiting patients with cardiovascular disease or diabetes with end-organ damage who are identified as completely ACEi intolerants [8]. These patients were randomized to Telmisartan 80 mg/day versus placebo. After median follow-up of 56 months, compared to placebo, Telmisartan reduced the composite of CV death, myocardial infarction, and stroke with OR 0.87, [95% CI 0.76–1.0] with unadjusted *p* value = 0.048. Worth mentioning that in that ACEi intolerant selected cohort, DC was comparable in the Telmisartan and placebo groups and had occurred in 21.6% versus 23.8%, respectively [8].

Although evidences are still insufficient to qualify Telmisartan as one of the standard RAASi in HFrEF unselected cohorts, it seems that selectively in HFrEF and CKD patients Telmisartan is better tolerated, leads to fewer WRF, shows less frequent drug interruptions, and achieves higher RAASi doses. Such superior and consistent inhibition of RAAS proved to be paralleled by greater clinical benefits that what can be achieved by low-doses of frequently interrupted other standard ACEi/ARBs.

### Limitations

This randomized study has some limitations to be disclosed. The relatively small sample size (107), and the short period of follow-up might have led to underestimation of the endpoints. Also, having an open-label design would allow for potential interpretation bias of the NYHA class. Unfortunately, the logistic setup of the recruiting center could not allow for a double-blinded design, nevertheless, the investigators added the 6MWT as an objective assessment tool to offset such concern. Being self-funded, the available resources did not permit to perform baseline and repeated testing of NT-pro-BNP and albumin/creatinine ratio (ACR) for all patients, while they could have added a critical quantitative prognostic tool to assess study drug performance. A larger, double-blinded, multi-center study, designed with a longer follow-up and entailing BNP, ACR and LV strain assessment in baseline and by the study end is strongly recommended to confirm the findings of this study

## Conclusions

In HFrEF patients with concomitant moderate CKD, Telmisartan showed better tolerability-, sustainability- and safety-profiles compared to the conventional RAASi, Enalapril. Because of ability to sustain higher RAASi dose with less frequent WRF and DR/DC, Telmisartan improved NYHA class changes, reduced HFH and improved effort tolerance by 6-months compared to Enalapril.

## Data Availability

Data can be provided (anonymized) upon reasonable request sent to the corresponding author.

## Abbreviations

6MWT: Six-minute walk test
ACEi: Angiotensin converting enzyme inhibitor
ARB: Angiotensin receptor blocker
ARNI: Angiotensin receptor neprilysin inhibitor
b.i.d: Twice daily
BB: Beta blocker
BNP: Brain Natriuretic peptide
BP: Blood pressure
CI: Confidence interval
CKD: Chronic kidney disease
CV: Cardiovascular
CVD: Cardio-vascular disease
DC: Dis-continuation
DR: Dose reduction
eGFR: Estimated glomerular filtration rate
ESKD: End-stage kidney disease
GDMT: Guidelines directed medical therapy
HF: Heart failure
HFH: Heart failure re-hospitalization
HFrEF: Heart failure with reduced ejection fraction
IQR: Inter-quartile range, denoting the 25th and 75th percentiles
LV: Left ventricle
LVEF: Left ventricular ejection fraction
MDRD: Modification of Diet in Renal Disease
mg: Milligrams
MRA: Mineralocorticoid antagonist
NYHA: New York Heart Association
o.d: Once daily
OR: Odds ratio
PPAR-g: Peroxisome Proliferator Activated Receptor-Gamma
RAAS: Renin angiotensin aldosterone system
RAASi: Renin angiotensin aldosterone system inhibitor(s)
SGLT2i: Sodium Glucose co-Transporter type-2 inhibitor
TTE: Transthoracic echocardiography
WRF: Worsening renal function
